# Evaluating the feasibility of a nurse-led self-management support intervention for kidney transplant recipients: a pilot study

**DOI:** 10.1186/s12882-019-1300-7

**Published:** 2019-04-27

**Authors:** Janet M. J. Been-Dahmen, Denise K. Beck, Mariëlle A. C. Peeters, Heleen van der Stege, Mirjam Tielen, Marleen C. van Buren, Erwin Ista, AnneLoes van Staa, Emma K. Massey

**Affiliations:** 1 Research Center Innovations in Care, Rotterdam University of Applied Sciences, P.O. Box 25035, 3001 Rotterdam, HA the Netherlands; 2 Department of Internal Medicine – Section Nephrology & Transplantation, Erasmus MC University Medical Center, P.O. Box 2040, 3000 Rotterdam, CA the Netherlands; 3 Department of Internal Medicine – Section Nursing Science, Erasmus MC University Medical Center, P.O. Box 2040, 3000 Rotterdam, CA the Netherlands; 4 Erasmus School of Health Policy & Management, Erasmus University Rotterdam, P.O. Box 1738, 3000 Rotterdam, DR the Netherlands; 5 Intensive Care Unit, Erasmus MC University Medical Center-Sophia Children’s Hospital, P.O. Box 2060, 3000 Rotterdam, DR the Netherlands

**Keywords:** Organ transplantation, End-stage-renal disease, Self-care, Self-management support, Nurse practitioner, Nursing, Intervention evaluation, Pilot study

## Abstract

**Background:**

To support effective self-management after kidney transplantation, a holistic nurse-led self-management support intervention was developed using the Intervention Mapping approach. The primary aim was to evaluate the feasibility, acceptability and fidelity of the intervention for kidney transplant recipients and professionals. The secondary aim was to explore preliminary effects on outcomes.

**Methods:**

A pilot study was conducted in 2015–2017 to evaluate the intervention. Nurse Practitioners (NP) guided recipients in assessing 14 life areas using the Self-Management Web. Participants were supported in developing self-regulation skills which can be applied to self-management of the illness. Strategies included goal setting, action planning, and promotion of motivation and self-efficacy. Adult recipients from an outpatient clinic of a Dutch University Hospital who underwent their transplant at least 1 month ago, were invited to participate. NPs, nephrologists and recipients were interviewed to assess feasibility, fidelity and implementation experience. Consultations were videoed and analysed to assess fidelity. To assess the preliminary effects, the intervention group completed baseline (T0) and follow-up (T1) questionnaires on self-management behavior, self-efficacy, quality of life and quality of care. A historical control group of kidney transplant recipients completed the same questionnaires at T1.

**Results:**

Twenty-seven recipients agreed to participate in the intervention group, of which 24 completed the intervention and 16 completed baseline and follow-up surveys. The control group consisted of 33 recipients. Professionals and recipients appraised the open, holistic focus of the intervention as a welcome addition to standard care and felt that this helped to build a relationship of trust. Recipients also felt they became more competent in problem-solving skills. The within-group analysis showed no significant increase in patients’ self-management skills. The between-groups analysis showed significantly higher medication adherence among the intervention group (*P* = 0.03; G = 0.81). The within-groups analysis showed a significantly higher perceived quality of care (*P* = 0.02) in the intervention group.

**Conclusion:**

This holistic nurse-led self-management support intervention was found to be feasible and acceptable by professionals and recipients alike. This pilot had a small sample therefore further research is needed into the potential effects on self-management behavior and well-being of transplant recipients. ISRCTN Trial Registry: ISRCTN15057632 (registered retrospectively on 20-07-2018).

**Electronic supplementary material:**

The online version of this article (10.1186/s12882-019-1300-7) contains supplementary material, which is available to authorized users.

## Background

Kidney transplantation is the preferred treatment for patients with end-stage renal disease because of better quality of life and survival compared to dialysis [1, 2]. After transplantation, recipients need to learn to adapt to lifestyle recommendations, the medication regimen, changing social roles and emotional challenges [3, 4]. As patients live longer with chronic conditions and often multiple comorbidities, there is an increasing focus on effective self-management and optimizing quality of life [5, 6]. Self-management has been defined as managing the medical, emotional and social challenges of a chronic condition in daily life with the aim of achieving optimal quality of life [7]. Optimal self-management can indirectly improve the quality of life of kidney transplant recipients [8].

One of the core tasks of nurses and nurse practitioners is to support self-management in the post-transplant period, and by doing so promote optimal medical and psychosocial outcomes [9, 10]. In the post-transplant period, self-management support interventions often focus on promoting recipients’ medication adherence and self-monitoring through information provision [11–14], even though it is well known that providing information is not enough to change behavior [15]. This narrow focus neglects the psychological and social challenges reported by recipients [16, 17]. Research has shown that nurses tend to overlook recipients’ social and emotional challenges and the importance of promoting skills to cope with them [10, 18, 19]. Key element of nurses’ self-management support should be coaching recipients to develop problem-solving skills and self-confidence [15]. Support focusing on people’s intrinsic motivation and self-efficacy seems to be effective to ensure persistence and performance of new behavior [15, 20]. However, holistic and tailored SMS interventions for kidney transplant recipients are scarce. In order to meet the needs of kidney transplant recipients, a holistic nurse-led SMS intervention was developed using the Intervention Mapping approach [21].

The primary aim of this initial pilot study was to gain insight into the feasibility, acceptability and fidelity of a nurse-led self-management (support) intervention for kidney transplant recipients (process evaluation). The secondary aim was to make a preliminary assessment of the effects of this intervention on self-management behavior, self-efficacy, quality of life and quality of care (effect evaluation). Table 1 provides an overview of the research questions.

**Table 1:** Research questions and data-collection methods

Research questions	Data-collection techniques				
	Quantitative	N intervention group (T0/ T1)	N control group (T1)	Qualitative	N
*1. To what extent did the NPs carry out the SMS intervention as described in the protocol? (fidelity)*	Therapy Adherence Measurement (TAM- score)	16^a^		Observations	6
*2. What are the experiences of recipients and professionals regarding the applicability, usability and acceptability of the nurse-led self-management intervention? (feasibility)*	Questionnaire to rate areas recipients perceived to be important and which areas were addressed during the consultation with the nurse	16	33	Individual interviews with patients	11
				Individual interviews with Nurse Practitioners	2
				Individual interviews with doctors	2
*3. What are the differences in primary and secondary outcomes of recipients within the intervention group?*	Questionnaire (T0-T1)	16			
*4. What are the differences in primary and secondary outcomes between recipients in the control and intervention group?*	Questionnaire (T1 intervention –control)	16	33		

^a^Only measured at T1

## Methods

### Study design

A pilot study with a mixed-methods design was conducted. As nurses were trained in communication techniques prior to implementation, we deemed it impossible for them to withhold these skills for a potential control group. Therefore A historical control group was used.

### Sample and participants

#### Intervention group

##### Recipients

A total population sampling approach was used to select kidney transplant recipients aged 18 years and older, who had a functioning graft and underwent their transplant one to 8 months ago. Recipients who visited the outpatient post-transplantation clinic and were in follow-up by one of the participating nurse practitioners at a Dutch University Hospital, between December 2015 and September 2016, were invited to participate. Recipients with cognitive limitations, acute psychiatric problems, who did not speak the Dutch language, with more than two previous consultations with a NP after their transplantation, who underwent treatment in isolation, participated in other studies, or who were undergoing dialysis or were expected to start with dialysis within 3 months were excluded. No limitations were set to the type of donor or prior renal replacement therapy. All participants were being treated according to a standard protocol. Most newly transplanted patients start on a standard regimen of tacrolimus, mycophenolate mophetil acid (MMF), and prednisone for the first 6 months. A purposive selection of recipients, selected in order of completion of the intervention, were asked to participate in an individual interview and/or observation.

##### Professionals

The two nurse practitioners (NPs) who held post-transplant consultations and nephrologists with whom they work in the post-transplant outpatient clinics were invited to participate in the interviews to evaluate implementation of the intervention.

#### Control group

Data from the historical control group was collected prior to nurse training and implementation of the intervention. A total sampling approach was used to select recipients who visited the outpatient post-transplantation clinic of a Dutch University Hospital and who were transplanted between 5 and 12 months earlier. The inclusion and exclusion criteria were the same as those of the intervention group.

### Nurse-led self-management intervention

The intervention was developed using the Intervention Mapping approach [21]. First, recipients’ and nurses’ needs were assessed through individual interviews and focus group, an observational study, a realist review, a qualitative synthesis, and a Q-methodological study [15, 17–19, 22–24] (step 1). Subsequently, change objectives for the self-management support intervention were formulated (step 2). In step 3, theory-based intervention methods were selected and translated into practical implications. Theoretical guidance came from the Self-regulation Theory [25], techniques from Motivational Interviewing [26], and Solution-Focused Brief Therapy (SFBT) [27]. Thereafter, the intervention protocol, training syllabus, implementation checklist and the Self-management Web were developed (step 4). Finally, the intervention was implemented in 2015 (step 5)*.* The intervention was called ZENN, an acronym derived from the Dutch translation of Self-Management After Kidney Transplantation (ZElfmanagement Na Niertransplantatie). A full description of the intervention development is available elsewhere [28].

**Fig. 1:**
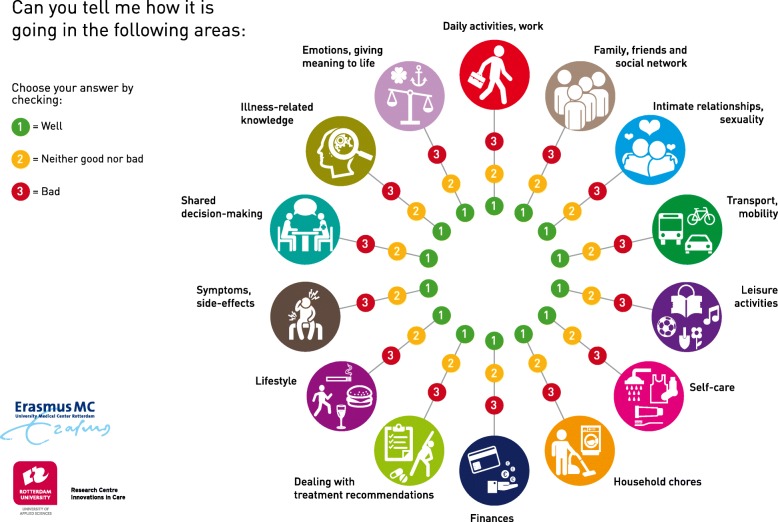
Self-management Web

The following key elements were included in the intervention: opportunities for tailoring within a general structure; assessment of patients’ needs and preferences using a holistic approach; principles of shared-decision making; and patient empowerment. The overall goal was to enhance recipients’ self-management skills in order to integrate treatment and life goals and subsequently optimize recipients’ quality of life and health-related outcomes. The steps of the intervention were divided over four sessions. In the first session, self-management challenges were assessed with a Self-Management Web (Fig. 1), specifically designed for this purpose. This visual communication aid offers an overview of 14 life areas (e.g. work, emotional well-being, sexuality, and transport and mobility), thereby adding structure to the consultation and widening the range of topics discussed. Recipients evaluate each area by indicating whether they are doing well (1 = green), neither good /nor bad (2 = orange) or bad (3 = red). Once the challenges had been identified by the recipient, the NPs employed solution-focused communication techniques to discuss desired outcomes, self-efficacy, to set SMART-goals and make an action plan. Progression towards goal attainment and outcome expectations were discussed in the second and third session. Goal progress, relapse prevention and generalization of learned skills to other challenges were discussed in the fourth session. Over the course of these sessions NPs and recipients re-assessed the original 14 life areas to detect other emerging issues and assess priorities.

During the intervention, double appointments were made for recipients (30 min rather than 15 min) with the NPs at the outpatient clinic. Time between the sessions ranged from 2 weeks to several months, depending on frequency of standard care appointments. If the period between session 1 and 2 was over a month, a telephone consult with the NP was scheduled.

Two NPs received two half-day training sessions, an intervention protocol and a booster session during which problems encountered could be discussed and techniques practiced. An experienced psychotherapist (AvtS) and a psychologist (DB) provided the training.

### Data-collection

Table 1 provides an overview of the data-collection methods per research question.

#### Qualitative data

In order to analyse fidelity, six consultations were video recorded (sessions 2–4) and analysed using a semi-structured observation protocol (JB & DB) between September 2016 and November 2016. The first consultation was not filmed to avoid interrupting the process of building trust between the NP and recipient.

To assess applicability, usability and acceptability, semi-structured interviews with recipients, NPs, and nephrologists were conducted by JB, DB and EI between September 2016 and March 2017. Recipients who completed the intervention were purposefully invited to participate in a semi-structured interview using an interview guide. Interview questions focused on: the holistic focus; intervention components; patient-activation; use of the intervention at home; and logistics. The interviews with professionals focused on barriers and facilitators of the intervention, intervention components, the holistic focus, NPs’ competency to deliver the intervention. All interviews were audio-recorded and transcribed.

#### Quantitative data

Baseline questionnaires were completed by the intervention group before the first session of the intervention (T0) and follow-up questionnaires were completed after the last session (T1). The control group completed the T1 questionnaire at a comparable moment to the intervention group (5–12 months after transplantation). The intervention group also filled in the therapy adherence measurement (TAM) questionnaire. JB or DB gave the questionnaires to recipients to complete either in the waiting room or at home.

### Outcome measures

Table 2 provides an overview of the outcome measures and questionnaires.

**Table 2:** Outcome measures and questionnaires

Primary outcome	Secondary outcomes	Questionnaire
Self-management knowledge and behaviour		- Partners in Health Scale (PIH) [29–31]
	Quality of life	- 36-Item Short Form Survey (SF-36) [32] - The World Health Organization Quality of Life - brief version (WHOQol-BREF) [33]
	Self-efficacy	- Visual Analogue Scales (VAS) - Self-Efficacy for Managing Chronic Disease 6-item Scale (SECD6) [16, 35]
	Feelings after kidney transplantation	- The Transplant Effects Questionnaire (TxEQ) [36, 37]
	Quality of nurse-led care	- American Consumer Assessment of Health Plan Surveys (CAHPS) [38–40]
	Social support	- Health Education Impact Questionnaire (heiQ) [41]
	NPs’ fidelity to intervention protocol	- Therapy Adherence Measurement (TAM) [42, 44, 45]
	Importance vs actual attention to topic during nurse-led consultation session	- Self-developed questionnaire

The primary outcome of this study was recipients’ self-management knowledge and behaviour measured with the 12-item Partners in Health Scale [29–31]. Recipients scored on a 8-point Likert scale (where 1 indicates poor self-management and 8 good self-management) [31]. While the original Australian PIH had four subscales (α = .82), the Dutch version consists of two-subscales: 1) knowledge and coping; 2) recognition and management of symptoms, and adherence to treatment. The Cronbach’s alphas of the subscales were 0.80 and 0.72 respectively. The correlation between the subscales was 0.43 [31].

Secondary outcomes were quality of life, general health, self-efficacy, experienced pain and fatigue, responses of transplant recipients to receipt of an organ, quality of nurse-led care, social support, and NPs’ fidelity. Quality of life was assessed with the SF-36 (range score 0–100) [32]. Four subscales were used: role limitations due to physical health problems (RP), vitality (VT), role limitations due to emotional problems (RE), and general mental health (MH). A higher score indicates a better quality of life. The Cronbach’s alphas of the Dutch version for the four subscales RP, VT, RE and MH were, respectively, α = 0.88, α = 0.83, α = 0.83, and α = 0.86. Two questions of the World Health Organization Quality of life Instrument (WHOQol-Bref), validated in English [33], were used to measure recipients’ general quality of life: “How would you rate your quality of life” and “How satisfied are you with your health”. These questions had a 5-point Likert scale (1 indicating poor and 5 good quality of life) [34].

Self-efficacy was measured with the Self-Efficacy for Managing Chronic Disease 6-item scale (SECD-6) [16, 35]. Recipients rated on a 10-point Likert scale, with 1 indicating not at all confident and 10 total confidence. The Cronbach’s alpha of the English scale is α = 0.91 [16, 35]. Our research group translated the scale into Dutch, but it has not been validated.

Recipients scored their general health, pain and fatigue on a 10-point visual analogue scale (VAS). Higher scores indicated better health, more fatigue, or pain. To assess recipients’ responses to the receipt of an organ, The Transplant Effects Questionnaire (TxEQ) was used [36]. The TxEQ encompasses 23 items in five subscales: worries about the transplant, feelings of guilt towards the donor, disclosure about having a transplant, feelings and behaviour regarding medication adherence, and perceived responsibility to others [36]. Recipients scored items on a 5-point Likert scale (1 = strongly disagree to 5 = strongly agree). The Cronbach’s alphas of the Dutch version of the TxEQ range from 0.66 to 0.79 [37].

Recipients’ perceived quality of nursing care was measured with the subscale ‘patient-centeredness’ of the American Consumer Assessment of Health Plan Surveys (CAHPS). This subscale consists of 5 questions using a 4-point Likert scale (from 1 = no, definitely not to 4 = yes, definitely). The scale is validated for use in the Dutch context (α = 0.90) [38–40].

Social integration and support was measured with a subscale of the Health Education Impact Questionnaire (HEIQ) [41]. This subscale measuring social integration and support consist of 5 items scored on a 4-point Likert scale (1 = total disagree and 4 = total agree); Cronbach’s alpha is 0.86. Higher scores indicate high levels of social interaction, higher sense of support and seeking more support from others. Since our research group translated this subscale into Dutch, it has not yet been validated.

Delivering an intervention as intended, also referred to as fidelity [42], is positively associated with better outcomes [43]. NPs’ fidelity was measured with a self-developed Therapy Adherence Measurement (TAM). The development of the TAM was guided by characteristics as described in the literature [42, 44, 45]. First, the purpose of this fidelity measurement was established, after which essential elements of the intervention were identified an included in the measure (Additional file 1 provides the 16 questions of the TAM).

Our research group developed a questionnaire based on the areas of the Self-Management Web. Patients indicated the importance of paying attention to various topics and the actual attention NPs paid to these topics. This scale consists of 15 items scored on a 3-points Likert scale (importance questions: 1 = not important, 2 = somewhat important, and 3 = very important; attention questions: 1 = no attention, 2 = some attention, 3 = much attention). To be able to measure differences, answer options 1 and 2 were recoded as negative and 3 as positive.

### Data-analysis

#### Qualitative analysis

Interviews were transcribed verbatim and imported into Atlas.ti 7.0. Data-driven codes were assigned to text. The results of first coding were discussed in the research team (JB, EI & EM) until agreement was reached. Any disagreements were resolved by discussion. Thereafter, codes were sorted into categories and further refined during the coding process [46, 47].

Two researchers (JB & DB) independently observed the videoed consultation sessions using a predetermined observation list based on the essential elements of the intervention protocol. Results were compared, and differences were discussed.

#### Quantitative analysis

Medians, interquartile ranges (IQR) and proportions were used for descriptive analyses. The Wilcoxon test was used for the baseline - follow-up analysis within the intervention group (T0-T1) and the Wilcoxon test and Chi-square test were used for testing differences of the intervention and control group (T1-C). Effect sizes were calculated for the outcome measures with the bias-correct effect size Hedges (G). Effect sizes were interpreted as small (=0.20), medium (=0.50), or large (=0.80) [48]. IBM SPSS Statistics 24.0 was used for statistical analyses.

### Ethical considerations

Transplant recipients who were eligible for the study were informed about this study by their NP (MT and MB) and received an information letter. DB called recipients to ask whether the information was clear and they were willing to participate. Only those recipients who returned the signed informed consent form participated. An additional informed consent form was signed by recipients participating in the interviews or observations. After completion of the study, participants received a €10 gift voucher. All participants were assured of confidentiality: data were processed anonymously, and medical staff did not have access to the non-anonymous data. The study protocol was approved by the Medical Ethical Committee of the University Medical Center Rotterdam (MEC-2015-317).

## Results

**Fig. 2:**
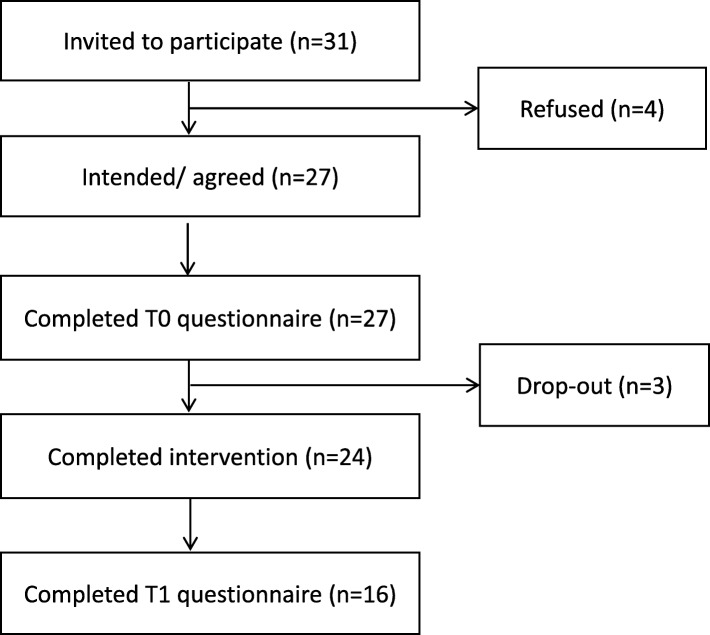
Flow chart of participants in the intervention group

Thirty-one kidney transplant recipients were invited to participate in the intervention group, of which 27 agreed to participate but only 24 went on to complete the intervention. Figure 2 shows the flowchart of the kidney transplant recipients in the intervention group. There were no significant differences between the results of recipients who underwent two or four sessions. For the control group, 48 recipients were invited to participate, 33 returned the questionnaire.

Sample characteristics are shown in Table 3. There were no significant differences between the characteristics of the control group, intervention group and non-responders of the intervention group.

**Table 3:** Sample characteristics

Characteristics	Control group (*n* = 33)	Intervention group (*n* = 24)
Age (median; IQR)	59.8; IQR 29.4–75.8	59.7; IQR 28.7–72.2
Gender		
Male (n; %)	22 (66.7)	17 (70.9%)
Marital status		
Married/ living together (yes; n; %)	21 (63,6)	11 (46.9%)^b^
In paid employment yes; (n; %)	10 (30.3)	9 (37.5%)
Highest educational attainment (n; %)	^a^	^b^
None	1 (3.0)	1 (4.2%)
Primary school	3 (9.0)	1 (4.2%)
Secondary School	9 (27.3)	7 (29.1%)
Higher education	18 (54.5)	12 (50%)
Number of transplantations (n; %)	^a^	^b^
1	26 (78.8)	21 (87.5)
2	4 (12.1)	3 (12.5)
3	3 (9.1)	0 (0)
Ethnicity (n; %)	^a^	
African	3 (9.1)	5 (20.8)
Asian	4 (12.1)	2 (8.3)
European	22 (66.8)	16 (66.7)
South American	0 (0)	1 (4.2)
Turkish	2 (6.0)	0
Dialysis before transplantation (n; %)		
Yes	21 (63.6)	17 (70.8)
No	12 (36.4)	7 (29.2)
Comorbidity (n; %)		
Diabetes	9 (27.3)	6 (25.0)
Cardiac Event	6 (18.2)	5 (20.8)
CVA event	5 (15.2)	1 (4.1)
Vascular Event	3 (9.1)	1 (4.1)

^a^missings (*n* = 2)

^b^missings (*n* = 3)

### Research question 1: to what extent did the NPs carry out the self-management support intervention as described in the protocol? (fidelity)

#### Fidelity

Fifteen recipients completed the Therapy Adherence Measurement (TAM). Nine recipients discussed non-medical topics with their NP. Key elements of the protocol e.g. use of the Self-Management Web, goal setting, action planning, self-efficacy, and motivation were reported to be addressed by three quarters of the recipients. Recipients reported the focus on the sessions to be more on problems than solutions. Data are presented in Additional file 1.

#### Observations of the consultations

Six consultation sessions were filmed and analyzed. In general, all intervention steps were completed and the communication techniques of Motivational Interviewing and Solution-Focused Brief Therapy were used. For example, recipients were asked about their motivation and confidence in pursuing their goal: *“How much confidence do you have in your ability to improve this?”* (NP2). The next session was started by referring to what had been discussed previously. NPs discussed recipients’ progress on their goal attainment plan, usually by asking recipients to rate their progress on a scale from 0 to 10. If recipients had not attained their goals, NPs praised recipients for their efforts and discussed the experienced barriers. NPs used the solution-focused approach to stimulate recipients in a positive way:

*“Given the fact that you’ve been ill in the meantime, you’ve actually done really well. Really good that you’ve doing more, because your fitness had been improving every time.” (NP1)*

Alternative strategies for goal attainment were also discussed. Sometimes, recipients set unattainable goals for the next session. Then NPs helped them to reformulate these into small and realistic steps.

Both NPs were able to tailor the intervention to their recipient’s specific needs, however some aspects of the protocol were more challenging: for example, asking open questions and encouraging recipients to develop their own solutions instead of offering potential solutions.

### Research question 2: what are the experiences of recipients and professionals regarding the applicability, usability and acceptability of the nurse-led self-management intervention? (feasibility)

#### Recipients’ experiences

Eleven recipients, proportional to the distribution of recipients across the NPs, participated in an interview about their experiences with the intervention.

#### Need for holistic support

The opportunity to discuss emotional and social issues during outpatient consultations with a NP was highly appreciated by recipients. In standard care, they had experienced that healthcare professionals focus on medical issues leaving little time to discuss other topics. One recipient explained the benefit her experience:

“*The first few times I thought, does this make sense? After two or three times we discussed more serious [topics]. We had deep, long conversations. These helped me. It was not just nice small talk. We talked about feelings……This helped me. It helped me to become active.” (R2)*

Most recipients expressed that the intervention should be made available for all kidney transplant recipients. One recipient stated no personal need for this holistic support, as he did not wish to discuss personal matters with his doctor or NP. Still, he felt that the intervention could be beneficial for others.

In the T1 questionnaire, recipients were asked to rate which areas they perceived to be important and which areas were addressed during the consultation with the NP. Recipients in the intervention group rated the importance of sexuality (*P* = 0.016), leisure activities (*P* = 0.036), adjusting lifestyle (*P* = 0.038), psychological well-being (*P* = 0.003), dealing with lack of understanding of others (*P* = 0.015); and (re)initiating normal life (*P* = 0.030) significantly higher than recipients in the control group. No significant differences were measured within the intervention group between baseline and follow-up.

At T0, there was a discrepancy between patient-reported areas of importance and these topics being addressed (whereby important topics were not being discussed) in the following areas: social context and relationships; sexuality; personal care; psychological well-being; dealing with lack of understanding of others; and (re)initiating normal life. There was a significant increase within the intervention group in the extent to which important areas were addressed by the healthcare professional: psychological well-being (*P* = 0.021), (re)initiating normal life (*P* = 0.046), being in control with own treatment process (P = 0.046), and dealing with the chronic condition (*P* = 0.025). These areas were addressed significantly more often in the intervention group than in the historical control group (Table 4).

**Table 4:** Importance of paying attention and actual attention paid to various topics

Topics	Answer options	Area perceived to be important (N; %)					Answer options	Area of importance addressed by NP (N; %)				
		T0	T1	C	*P*-value – Wilcoxon test (T0-T1)	*P*-value – Chi square (C-T1)		T0	T1	C	*P*-value – Wilcoxon test (T0-T1)	*P*-value – Chi square (C-T1)
(Unpaid) work or education	Not important	7 (46.7)	5 (31.3)	15 (50.0)	0.380	0.384	Not addressed	7 (53.9)	4 (26.7)	19 (63.3)	0.083	0.012
	Important	8 (53.3)^a^	11 (68.8)	15 (50.0)^c^			Addressed	6 (46.1)^c^	11 (73.3)^a^	11 (36.7)^c^		
Social contacts and relationships	Not important	3(20.0)	4 (25.0)	13 (43.3)	0.763	0.239	Not addressed	6 (46.1)	4 (26.7)	16 (51.6)	0.052	0.000
	Important	12 (80.0)^a^	12 (75.0)	17 (56.7)^c^			Addressed	7 (53.9)^c^	11 (73.3)^a^	15 (48.4)^b^		
Sexuality	Not important	8 (53.3)	5 (31.3)	21 (70.0)	0.166	0.016	Not addressed	9 (69.2)	6 (40.0)	27 (87.1)	0.096	0.002
	Important	7 (46.7)^a^	11 (56.8)	9 (30.0)^c^			Addressed	4 (30.8)^c^	9 (60.0)^a^	4 (12.9)^b^		
(Adjustment of) leisure activities	Not important	0 (0.0)	2 (12.5)	12 (40.0)	0.527	0.036	Not addressed	2 (16.7)	1 (6.3)	17 (54.8)	0.257	0.002
	Important	15 (100.0)^a^	14 (87.5)	18 (60.0)^c^			Addressed	10 (83.3)^d^	15 (93.7)	14 (45.2)^b^		
Practical matters in daily life (e.g. household)	Not important	4 (26.7)	3 (18.8)	13 (41.9)	0.132	0.088	Not addressed	3 (23.1)	3 (20.0)	21 (72.4)	0.564	0.001
	Important	11 (73.3)^a^	13 (81.2)	18 (58.1)^b^			Addressed	10 (76.9)^c^	12 (80.0)*	8 (27.6)^d^		
Transport and mobility	Not important	5 (33.3)	6 (37.5)	16 (53.3)	0.803	0.455	Not addressed	5 (28.5)	5 (33.3)	21 (72.4)	0.705	0.009
	Important	10 (66.7)^a^	10 (62.5)	14 (46.7)^c^			Addressed	8 (61.5)^c^	10 (66.7)*	8 (27.6)^d^		
Personal care (e.g. washing, dressing)	Not important	7 (46.7)	5 (31.3)	13 (43.3)	0.160	0.671	Not addressed	8 (57.1)	5 (31.3)	20 (66.7)	0.057	0.005
	Important	8 (53.3) ^a^	11 (68.7)	17 (56.7)^c^			Addressed	6 (42.9)^b^	11 (68.7)	10 (33.3)^c^		
Adjusting lifestyle (e.g. nutrition, exercise)	Not important	2 (20.0)	1 (6.3)	9 (30.0)	0.527	0.038	Not addressed	4 (30.8)	2 (12.5)	11 (37.9)	0.107	0.009
	Important	13 (80.0)^a^	15 (93.7)	21 (70.0)^c^			Addressed	9 (69.2)^c^	14 (87.5)	18 (62.1)^d^		
Psychological well-being	Not important	1 (6.7)	2 (12.5)	7 (23.3)	0.783	0.003	Not addressed	5 (35.7)	2 (12.5)	16 (55.2)	0.021	0.003
	Important	14 (93.3)^a^	14 (87.5)	23 (76.7)^c^			Addressed	9 (64.3)^b^	14 (87.5)	13 (44.8)^d^		
Dealing with the chronic condition	Not important	1 (6.7)	1 (6.7)	3 (9.7)	0.564	0.167	Not addressed	2 (14.3)	0 (0.0)	11 (37.9)	0.025	0.001
	Important	14 (93.3)^a^	14 (93.3)^a^	28 (90.3)^b^			Addressed	12 (85.7)^b^	16 (100.0)	18 (62.1)^d^		
Dealing with lack of understanding of others	Not important	5 (33.3)	2 (13.3)	10 (32.3)	0.119	0.015	Not addressed	9 (64.3)	5 (33.3)	21 (72.4)	0.053	0.002
	Important	10 (66.7)^a^	13 (86.7)^a^	21 (67.7)^b^			Addressed	5 (35.7)^b^	10 (66.7)^a^	8 (27.6)^d^		
(re)inting normal life	Not important	1 (6.7)	2 (12.5)	3 (9.7)	1.00	0.030	Not addressed	3 (21.4)	1 (6.3)	12 (40.0)	0.046	0.000
	Important	14 (93.3)^a^	14 (87.5)	28 (90.3)^b^			Addressed	11 (78.6)^b^	15 (93.7)	18 (60.0)^c^		
Medical issues around the condition	Not important	2 (13.3)	1 (6.3)	2 (6.4)	0.564	0.922	Not addressed	2 (14.3)	0 (0)	2 (7.1)	0.157	0.022
	Important	13 (86.7)^a^	15 (93.7)	29 (93.6)^b^			Addressed	12 (85.7)^b^	16 (100.0)	26 (92.9)^e^		
Referral to other health care professionals (if needed)	Not important	5 (33.3)	1 (6.3)	1 (3.1)	0.480	0.446	Not addressed	1 (7.2)	1 (6.3)	8 (26.7)	0.083	0.044
	Important	10 (66.7)^a^	15 (93.7)	31 (96.9)^a^			Addressed	13 (92.9)^b^	15 (93.7)	22 (73.3)^c^		
Being in control with own treatment process	Not important	1 (6.7)	0 (0.0)	1 (3.1)	0.480	0.216	Not addressed	1 (7.2)	1 (6.3)	10 (35.3)	0.046	0.002
	Important	14 (86.7)^a^	16 (100.0)	31 (96.9)^a^			Addressed	13 (92.9)^b^	15 (93.7)	20 (66.7)^c^		

^a^ missing (*n* = 1)

^b^missings (*n* = 2)

^c^missings (*n* = 3)

^d^missings (*n* = 4)

^e^missings (*n* = 5)

#### Evaluation of the intervention components

The Self-Management Web was rated as helpful and understandable, particularly the pictograms. Recipients felt invited to discuss a wide range of life areas with their NP, including topics they would never have thought about to discuss (e.g. financial problems or sexuality). Evaluating and assessing if recipients are doing well on the various life domains helped them to gain an overview of their progress after transplantation:

*“Well, the difference between the beginning and the end was quite spectacular. In the beginning, I had o lot of domains scored as bad. But at the end, I also had some good scores. Given that I still have medical issues, it was very nice for me to see that I made progress.” (R6)*

When a life domain was scored as ‘bad’, this triggered them to think about possible causes and solutions. Recipients knew NPs could not resolve their problems, but being encouraged to set concrete and specific goals helped them to make a step forward. Some recipients mentioned that after the intervention, they had acquired more knowledge about their illness*.*

A relationship of trust was usually built over several sessions and facilitated in-depth personal discussions. Some recipients stated they had become more competent in problem-solving skills over time. Recipients recognized the importance of intrinsic motivation to work on personal goals:

*“It has to come from inside. Nobody else could do it for you.” (R1)*

The skills learnt may be useful in tackling future problems and issues in daily post-transplant life. Recipients reported being preoccupied with medical complications; they therefore preferred to receive the intervention once these issues had been resolved.

#### Professionals’ experiences

Two NPs and two nephrologists were interviewed about their experiences with the intervention.

#### Holistic focus

The ability to have broader conservations with recipients about their daily life was appreciated by both NPs. Before implementing the intervention they did not have a structured approach to guide the conversation about emotional and social issues:

*“I really liked this. Especially the Self-Management Web is a nice opening to start the conversation. Discussing all these topics helped me to create a complete picture of my recipients and to get insight into their problems” (NP1)*

Especially for newly transplanted recipients, NPs saw the intervention as a valuable addition to usual care. In their experience, many recipients struggle with emotional problems after transplantation such as guilt, anxiety and even depression. During the intervention, they were surprised that even recipients with many medical problems still wished to talk about emotional and social issues. At the same time, both NPs felt a strong responsibility for monitoring recipients’ medical situation. Therefore, they considered it very important to have still enough time to focus on medical aspects. For the nephrologists, it was of added value that NPs were able to address sensitive topics with the recipients that were not discussed with them. One of the nephrologists emphasized the added value of providing psychosocial support:

*“I think that this intervention has an added value for recipients’ quality of life. I do not think we get better functioning kidneys, but we will get better functioning recipients.” (Nephr1)*

The other nephrologist wondered where the boundaries are for post-transplant care and preferred a focus on improving recipients’ therapy adherence.

#### Evaluation of intervention components

NPs reported experiencing a learning curve and being challenged to adapt their methods of communication and way of interacting with patients. The intervention required them to shift their focus from problems to solutions and from offering solutions to stimulating patients to generate these themselves.

*“First, I was dreading it. I was expected to do something I was not used to. I had to get out of my comfort zone.” (NP1)*

The Self-Management Web was regarded a useful communication aid to assess issues. According to NPs, recipients felt comfortable to discuss daily life issues and aspects that NPs never discussed before, such as financial problems to visit the outpatient clinic. Still, NPs found it difficult to encourage recipients to set SMART goals and to prevent disappointment.

Sometimes it was a challenge to end their consult in time, especially during the first session when all life areas were evaluated. The intervention is designed to empower the recipient, which also entails that they select the topics to work on. This sometimes created a dilemma for the NP, when a patient did not select an issue that they considered as an area for change (for example: lifestyle). NPs also considered it difficult when a recipient expressed intrinsic motivation to change behavior without turning it into action:

*“Someone wished to stop smoking but did not quit. It this situation, it was very hard to say something positive or give him a compliment. At one point, I asked him whether it was the right moment for him to stop smoking. He said: ‘Yes, I really want to quit’. Still, he did not show any indication of doing so. I then started to focus on one of his other goals. But every time, he started to discuss he wished to quit.”* (NP2)

Some aspects of the intervention were reported to feel as somewhat unnatural or forced: for example, asking about recipients’ self-efficacy and discussing recipients’ motivation.

The NPs experienced the training as very helpful, particularly the role playing and discussing the filmed consultations sessions. Reinforcement and positive feedback helped them to improve their skills. After the training, both NPs felt competent to deliver the intervention.

The intervention has provided them tools to activate and support recipients in behavioral change. It also helped them to build a relationship of trust with their recipients.

### Research question 3 & 4: what are the differences in outcomes of recipients?

#### Primary outcome

There were no significant differences in recipients’ self-management knowledge and behavior (PIH) within the intervention group (T0 vs T1) and between the intervention and control group (T1 vs C) (Table 5).

**Table 5:** Medians, interquartile ranges and *p*-values of the primary and secondary outcomes

Outcomes	Subdomain (questionnaire)	T0^a^		T1^a^		C^a^		P-value (Wilcoxon test)		Effect Size	
		N	Median (IQR)	N	Median (IQR)	N	Median (IQR)	C-T1	T0-T1	C-T1	T0-T1
Self-management knowledge and behaviour	Total Score (PIH^b^)	15	88.0 (81.0–92.0)	15	88.0 (81.0–94.0)	32	85.5 (80.0–93.0)	0.54	0.41	0.20	0.24
Self-management knowledge and behaviour	Knowledge and coping (PIH^b^)	15	51.0 (41.0–53.0)	15	51.0 (45.0–55.0)	33	47.0 (42.0–54.5)	0.47	0.43	0.23	0.27
Self-management knowledge and behaviour	Recognition and management of symptoms, adherence to treatment (PIH^b^)	15	38.0 (36.0–40.0)	15	38.0 (36.0–40.0)	32	38.5 (36.0–40.0)	0.89	0.69	0.18	0.09
Quality of Life	General quality of life (WHOQol-BREF^b^)	16	4.0 (3.0–4.0)	16	4.0 (3.0–4.0)	32	4.0 (3.0–4.0)	0.73	0.56	0.20	0.22
Quality of Life	Satisfaction with health (WHOQol-BREF^b^)	16	3.0 (2.0–4.0)	16	3.5 (3.0–4.0)	33	4.0 (3.0–4.0)	0.27	0.40	− 0.31	0.11
Quality of Life	Role limitations due to physical health problems (SF-36^b^)	15	0.0 (0.0–50.0)	15	75.0 (0.0–100.0)	30	87.5 (0.0–100.0)	0.78	0.02	− 0.11	0.78
Quality of Life	Role limitations due to emotional problems (SF-36^b^)	14	100.0 (0.25–100.0)	15	100.0 (50.00–100.0)	29	100.0 (66.7–100.0)	0.55	0.73	− 0.17	0.14
Quality of Life	Vitality (SF-36^b^)	16	50.0 (26.3–58.8)	16	32.5 (25.8–52.5)	32	42.5 (25.0–53.8)	0.58	0.03	− 0.14	−0.41
Quality of Life	General mental health (SF-36^b^)	16	75.5 (61.5–88.0)	16	84.0 (70.5–92.0)	32	87.5 (64.0–92.0)	0.75	0.27	0.11	0.27
Self-efficacy	Total score (SECD6^b^)	16	5.3 (3.2–7.2)	16	5.3 (2.8–7.6)	30	5.6 (3.8–7.3)	0.94	0.32	−0.04	0.20
Self-efficacy	VAS - health^b^	16	60.1 (32.6–81.1)	16	66.9 (50.7–79.8)	32	70.5 (53.8–80.6)	0.70	0.25	− 0.13	0.31
Self-efficacy	VAS -pain^b^	16	8.3 (2.5–36.0)	16	3,6 (0.4–25.7)	33	2.9 (0.7–14.0)	0.99	0.90	0.05	0.20
Self-efficacy	VAS - fatigue^b^	16	57.2 (9.4–74.5)	16	25.7 (19.6–65.5)	33	43.9 (8.6–61.9)	0.82	0.45	− 0.08	0.20
Transplant effects	Worry about the transplan (TxEQ^b^)	14	3.4 (2.5–4.0)	16	3.1 (2.6–3.5)	29	3.3 (2.7–3.7)	0.69	0.69	0.11	0.48
Transplant effects	Guilt towards the donor (TxEQ^b^)	16	2.1 (1.8–2.6)	16	2.0 (1.6–2.2)	33	2.2 (1.8–2.8)	0.08	0.07	0.54	0.34
Transplant effects	Disclosure about the transplantation (TxEQ^b^)	16	5.0 (4.0–5.0)	16	4.8 (4.4–5.0)	32	4.8 (3.7–5.0)	0.85	0.07	− 0.01	0.31
Transplant effects	Adherence to immunosuppressive medications (TxEQ^b^)	16	4.8 (4.4–5.0)	16	5.0 (4.6–5.0)	32	4.8 (4.1–5.0)	0.03	0.12	0.81	0.43
Transplant effects	Responsibility towards others (TxEQ^b^)	16	3.5 (3.0–4.0)	16	3.6 (3.3–4.0)	33	3.8 (3.0–4.3)	0.86	0.36	0.02	0.20
Social support	Social integration and support (heiQ^b^)	16	18.5 (13.5–20.0)	16	19.0 (15.0–20.0)	33	18.0 (15.0–20.0)	0.78	0.91	− 0.09	−0.04
Patient-centred care	Total score (CAHPS^b^)	15	19.0 (18.0–20.0)	15	20.0 (20.0–20.0)	32	19.5 (18.0–20.0)	0.05	0.02	0.56	0.56

^a^*C* Control group, *T0* Intervention group at baseline, *T1* Intervention group after receiving the intervention

^b^*PIH* Partners in health scale, *WHOQol-BREF* The world health organization quality of life - brief version, *SF-36* 36-item short form survey, *SECD6* Self-efficacy for managing chronic disease 6-item scale, *VAS* Visual analogue scales, *TxEQ* The transplant effects questionnaire, *heiQ* Health education impact questionnaire, *CAHPS* American consumer assessment of health plan surveys

#### Secondary outcomes

No significant differences in quality of life between the intervention and control group were measured with the SF-36. However, recipients within the intervention group reported a significantly higher Quality of life (*P* = 0.02) with a medium effect size (G = 0.78) on the domain Role limitations due to physical health problems compared to the control group. However, a significant lower Quality of life was reported on the domain Vitality (*P* = 0.03; G = -0.41). Further, no significant differences were found on the subdomains general quality of life and satisfaction with health on the World Health Quality of Life (WHOQol-Bref) questionnaire. No significant differences in self-efficacy within (*P* = 0.32; G = 0.20) and between groups (*P* = 0.94; G = -0.04) were found. There were also no significant differences in health, pain and fatigue (VAS-scores). A significant difference was found between the control group (median 4.8) and the intervention group (median 5.0) in self-reported adherence to immunosuppressive medication (P = 0.03; G = 0.81). The differences on the other subdomains of the TxEQ were not significant. The domain ‘guilt towards the donor’ was higher in the control group (G = 0.54). There was no significant difference on social integration and support within the intervention group (*P* = 0.91; G = -0.04) and between the intervention and control group (*P* = 0.78; G = -0.09).

The quality of patient-centered care provided by NPs improved significantly between baseline and follow-up in the intervention group (*P* = 0.02; Median T0 = 19.0 & T1 = 20.0), but no significant differences in quality of care were found between groups (C-T1). The effect size in both groups was medium (G = 0.56).

## Discussion

This pilot study was conducted to evaluate the feasibility of a newly developed, holistic, nurse-led, self-management intervention [28]. Although several self-management interventions for patients with various chronic conditions have been developed, interventions specifically for kidney transplant recipients are scarce and mostly focus on providing support for medication adherence [11, 13].

The qualitative findings of this study showed that our intervention is feasible and is promising to help kidney transplant recipients deal with post-transplant challenges. According to most professionals and recipients, the holistic focus of the intervention was a welcome addition to standard care. Prior to the intervention, professionals largely focused on medical support, and overlooked recipients’ need for emotional and social support [10, 17–19]. NPs were challenged to broaden their view and adapt to using solution-focused communication techniques.

While the quantitative findings of this pilot showed no significant changes in recipients’ self-management behavior, the within-group (T0-T1) analysis indicated a significant improvement in the quality of delivered patient centered care, and quality of life - physical role. Changes in recipients’ quality of life- physical role may be explained by gradual improvements in the medical situation and physical recovery after transplantation. After the intervention, this group reported significantly higher medication adherence than the control group. Before the implementation of the intervention, patients indicated that it was important for professionals to pay attention to psychosocial topics while these were not frequently addressed during consultations. After the intervention, significantly more attention was paid to these topics in the intervention group compared to the control group. This is an indication than the protocol was followed, and that patients’ needs were being more sufficiently addressed.

Discussing various areas of life with a NP, helped recipients to create awareness in the challenges they face and the progress they made during the intervention. After completing the intervention, recipients felt more competent in problem-solving skills, which should be confirmed by a more extensive investigation into potential effects on self-management behavior and well-being of transplant recipients. For persistence and performance of new behavior, it is important that recipients have the motivation and self-efficacy that they are capable to deal with various situations [15].

Tailoring was an essential component of the intervention. The need for tailoring can be explained by the variation in kidney transplant recipients attitude, needs and preferences towards self-management support [24]. A personal approach instead of an ‘one size fits all’ approach for support is desired. Chronically ill patients wish to be seen as individuals with personal needs [23]. Because various life areas were addressed using the Self-Management Web in the intervention, professionals were able to assess recipient’s challenges individually and to discuss solutions that were suitable for the individual recipient. The open assessment also enables recipients to bring forward their own ideas, needs and preferences, which is seen as an important part of self-management [5, 6]. It should be noted that not all recipients wish to receive holistic support [26] and that a high standard of care demands flexibility from the professional in altering their own style of delivery according to the patient’s preferences [49].

In complex interventions, the skills of health care professionals strongly influence the outcomes [50, 51]. For this reason, the NPs in this study were trained to perform the intervention, including booster sessions during implementation. Some aspects of the intervention were challenging to them, such as asking open questions and encouraging recipients to develop their own solutions rather than offering potential solutions. Respecting recipient’s autonomy in selecting life areas to focus on versus reaching optimal health outcomes is an ethical dilemma NP experienced when providing the self-management support intervention [52]. Nurses tend to support recipients to make the ‘right choices’ according to standard medical norms [52]. We emphasize that in order to address issues the medical staff feel important, they need to take the priorities of the patient seriously. The resulting positive relationship and learned meta-skills can help address other self-management challenges. Training in Solution-Focused Brief Therapy (SFBT) can influence nurses’ communication skills positively [53]. In this study NPs indicated that particularly receiving feedback about their skills in booster sessions helped them to become more competent in performing the SFBT.

In contrast to many self-management interventions [15], the intervention was developed according to a strong methodological procedure, including techniques of behavioural change that have a strong theoretical and evidence base. Strengths-based interventions such as those using SFBT and MI are promising in supporting recipients’ self-confidence [54]. Another strong point of this pilot study is the mixed-method design, which is recommended to evaluate complex interventions [55]. It helped us to gain insight into the various essential elements of the intervention: open assessment of recipients’ needs, holistic approach, tailoring advice, patient activation, building confidence and motivation, goal setting, solution focused, shared-decision making, and working on a relationship of trust between the patient and professional. These working mechanisms are in line with the tasks (*Assess, Advise, Agree, Assist, Arrange*) defined in the Five A’s model for health care professionals in self-management support [56]. In line with the aim to promote patient empowerment, the patient was in the lead and encouraged to set the agenda.

In future testing of self-management interventions, researchers should take into consideration that patient reported experiences are important. Paying attention to patient’s individual experiences increases the quality of care [57], which advocates for ‘context-based practice’ instead of evidence based practice [58]. Patients Reported Experience Measures (PREMs), such as the CAHPS questionnaire, are valuable to measure what kind of care is delivered and whether the patient was satisfied with this care (e.g. Did the nurse listen to you?). Such measures can be valuable additions in examining the effects of self-management interventions.

A limitation of this study is that the intervention was evaluated in a single-center, results may therefore not be generalizable to all kidney transplant recipients in other settings. This requires further investigation alongside the potential value for recipients of other organs. There are many challenges for daily living that are common for all chronically ill patients [59], therefore this self-management intervention might be suitable for patients with other transplanted organs or chronic conditions and their health-care professionals as well. Other limitations include the small sample size, which is inherent to a pilot study, and the fact that the intervention was not completely integrated into standard care. Future multi-center study, should also take note of any differences in pharmacological treatment between experimental and control groups.

## Conclusions

In conclusion, the nurse-led self-management support intervention we evaluated was found to be feasible and acceptable by professionals and recipients alike. Essential elements reported by professionals and recipients were: open assessment of recipients’ holistic needs, tailoring advice, patient activation, building confidence and motivation, goal setting, solution focused, shared-decision making, and building a relationship of trust between the patient and professional. This initial pilot had a small sample and a more extensive investigation is needed into the potential effects on self-management behavior and well-being of transplant recipients.

## Additional file

Additional file 1: This file shows the results of the Therapist Adherence Measure (TAM) questionnaire. In this questionnaire participants were asked questions on which essential elements of the intervention protocol had been carried out. The number of participants reporting each element is presented alongside percentages. (DOCX 14 kb)

## Abbreviations

AvtS: Adriaan van ‘t Spijker
CAHPS: The American Consumer Assessment of Health Plan Surveys
DB: Denise Beck
EI: Erwin Ista
EM: Emma Massey
HEIQ: Health Education Impact Questionnaire
IGR: Interquartile ranges
JB: Janet Been-Dahmen
MB: Marleen van Buren
MH: General mental health
MT: Mirjam Tielen
Nephr: Nephrologists
NP: Nurse practitioner
PIH: Partners in Health Scale
R: Recipient
RE: Role limitations due to emotional problems
RP: Role limitations due to physical health problems
SECD-6: Self-Efficacy for Managing Chronic Disease 6-item scale
SF-36: 36-item Short Form Survey
SFBT: Solution-Focused Brief Therapy
SMS: Self-management support
TAM: Therapy adherence measurement
TxEQ: The Transplant Effects Questionnaire
VAS: Visual Analoque Scale
VT: Vitality
WHOQol-Bref: The World Health Organization Quality of life Instrument
ZENN: ZElfmanagement Na Niertransplantatie
