# Supplementary material for: Evaluating the feasibility of a nurse-led self-management support intervention for kidney transplant recipients: a pilot study
Source: BMC Nephrol. 2019 Apr 27;20:143. doi: 10.1186/s12882-019-1300-7 (PMC6486974; doi:10.1186/s12882-019-1300-7)
Supplement: Supplementary file 1 — This file shows the results of the Therapist Adherence Measure (TAM) questionnaire. In this questionnaire participants were asked questions on which essential elements of the intervention protocol had been carried out. The number of participants reporting each element is presented alongside percentages. (DOCX 14 kb) [file 12882_2019_1300_MOESM1_ESM.docx]

**Additional file 1: Results of the TAM questionnaire**

|  | **Total (n = 15)**  **N (%)** |
| --- | --- |
| **Number of sessions followed**  *1*  *2*  *3*  *4*  *Others*  *6 sessions*  *I do not know* | 0 (0%)  0 (0%)  0 (0%)  12 (80.0%)  1 (6.7%)  1 (6.7%)  ^a^ |
| **How often were non-medical topics discussed?**  *Never*  *During 1 session*  *During 2 sessions*  *During 3 sessions*  *During 4 sessions* | 5 (33.3%)  1 (6.7%)  0 (0%)  3 (20.0%)  5 (33.3%)  ^a^ |
| **Topics discussed with NP**  *Daily activities*  *Social Network*  *Intimate relationships, sexuality*  *Transport, mobility*  *Leisure activities*  *Self-care*  *Household chores*  *Finances*  *Following treatment recommendations*  *Lifestyle*  *Symptoms, side-effects*  *Shared decision-making*  *Illness-related knowledge*  *Emotional and spiritual well-being* | 9 (60.0%)  8 (53.3%)  5 (33.3%)  2 (13.3%)  8 (53.3)  9 (60.0%)  10 (66.7 %)  2 (13.3%)  9 (60.0%)  7 (46.6%)  10 (66.7%)  6 (40.0%)  11 (73.3%)  8 (53.3%) |
| **How often was the Self-Management Web discussed?**  *Never*  *During 1 session*  *During 2 sessions*  *During 3 sessions*  *During 4 sessions* | 1 (6.7%)  4 (26.7%)  3 (20.0%)  1 (6.7%)  5 (33.3%)  ^a^ |
| **Did you receive the booklet for patients?**  *Yes*  *No* | 12 (80.0%)  3 (20%) |
| **How often did you set goals together with the NP?**  *Never*  *During 1 session*  *During 2 sessions*  *During 3 sessions*  *During 4 sessions* | 2 (13.3%)  3 (20.0%)  3 (20.0%)  2 (13.3%)  4 (26.7%)  ^a^ |
| **Did the NP discuss your motivation to set a goal?**  *Yes*  *No* | 13 (86.7%)  1(6.7%)  ^a^ |
| **How often did you create an action plan together with the NP?**  *Never*  *During 1 session*  *During 2 sessions*  *During 3 sessions*  *During 4 sessions* | 2 (13.3%)  2 (13.3%)  4 (26.7%)  1 (6.7%)  4 (26.7%)  ^b^ |
| **How often did the NP encourage you to make concrete when, why and with who you are gone work on a goal you set?**  *Never*  *During 1 session*  *During 2 sessions*  *During 3 sessions*  *During 4 sessions* | 3 (23.1%)  0 (0.0%)  1 (7.7%)  3 (23.1%)  6 (46.1%)  ^b^ |
| **Did the NP discuss your self-confidence to reach a goal?**  *Yes*  *No* | 12 (85.7%)  2 (14.3%)  ^a^ |
| **Did the NP check whether you made progress in the past period?**  *Never*  *During 1 session*  *During 2 sessions*  *During 3 sessions*  *During 4 sessions* | 2 (15.4%)  0 (0.0%)  4(30.7%)  7 (53.9%)  0 (0.0%)  ^b^ |
| **Did the NP discuss your satisfaction with your progress?**  *Yes*  *No* | 12 (85.7%)  2 (14.3%)  ^a^ |
| **If necessary, were your goals adjusted?**  *Yes*  *No*  *It was not necessary* | 10 (71.4%)  1 (7.1%)  3 (21.5%)  ^a^ |
| **Did the NP discuss with you how you can deal with problem in the future?**  *Yes*  *No* | 10 (71.4%)  4 (28.6%)  ^a^ |
| **What was the consultation with the NP focused on?**  *Solutions*  *Problems*  *I do not know* | 2 (15.4%)  8 (61.5%)  3 (23.1%)  ^b^ |

* ^a^ – missing (n=1); ^b^ – missings (n=2);
